# Novel Oncolytic Virus Armed with Cancer Suicide Gene and Normal Vasculogenic Gene for Improved Anti-Tumor Activity

**DOI:** 10.3390/cancers12051070

**Published:** 2020-04-25

**Authors:** Su-Nam Jeong, So Young Yoo

**Affiliations:** BIO-IT Foundry Technology Institute, Pusan National University, Busan 46241, Korea

**Keywords:** oncolytic virus, angiopoietin 1, TRAIL

## Abstract

Here, we developed a novel oncolytic vaccinia virus (NOV) with the dual advantages of cancer selectivity and normal vessel reconstructive activity by replacing the viral thymidine kinase (vTk) and vaccinia growth factor (VGF) genes with genes encoding TNF-related apoptosis-inducing ligand (TRAIL) and angiopoietin 1 (Ang1), respectively. The pan-cancer-specific oncolytic potency of NOV was confirmed in various human and mouse cancer cell lines (colon, liver, pancreas, cholangiocarcinoma, cervical cancer, osteosarcoma, and melanoma). Vaccinia virus (VV) treatment directly induced early apoptosis in tumors within 24 h, and this effect was enhanced with further engineering; VGF and Tk deletion with Ang1 and TRAIL insertion. Meanwhile, treatment with the conventional anti-cancer drug cisplatin did not induce apoptosis. A virus-treated CT26 mouse colon cancer syngeneic model showed attenuated tumor growth, which was in accordance with the results of percent survival measurement, CD8 expression analysis, and TUNEL staining with advanced genetic engineering (vAng1 < vTRAIL < NOV). Taken together, our results indicate that NOV induces cancer tissue apoptosis and anti-tumor immunity and may constitute a highly advantageous therapeutic agent for next-generation solid tumor virotherapy with pan-cancer-specific oncolytic activity and high biosafety.

## 1. Introduction

Oncolytic viruses (OVs) are currently used for cancer treatment, especially in cases of advanced cancers that are refractory to conventional therapies such as chemotherapy and radiotherapy. Oncolytic virotherapy (OVT), which is an effective technique that is distinct from other general gene therapies, utilizes OVs as molecular cancer therapeutic agents [1,2,3,4,5]. OVs are utilized excellently for this purpose; they are stable and clinically available because they selectively infect and replicate in cancer cells. Cancer selectivity and biosafety can be enhanced through OV genetic engineering, thereby providing unprecedented opportunities in the field of OVT [1,6]. Over the last 18 years, OVT research has dramatically increased [7]. As of 2018, 137 preclinical trials and 27 phase I trials were in progress, up 16% from 2017 [8]. Since Amgen’s FDA (Food and Drug Administration) approval of talimogene laherparepvec (T-VEC), clinical trials at SillaJen with pexastimogene devacirepvec (Pexa-Vec), a recombinant vaccinia virus, and other recombinant OVs have been initiated and are currently ongoing [2,9,10].

The main advantage of OVs is their infection, replication, and tumorlytic properties [11]. Cancer cells lysed by OVs secret antigens to attract immune cells to the tumor. Additionally, OVs have the potential to act on both primary and metastatic tumors [1,5,12]. While most OVs show limited activity in human cells, vaccinia virus (VV) exhibits high infectivity, oncolytic activity, and efficacy in both mice and human cells [13,14]. A member of the Poxviridae family, VV is a DNA virus with double-stranded DNA of ~190 kbp in length [14]. To date, Lister, Western Reserve, and Wyeth have been characterized as strains of VV. The safety and immunogenicity of this virus were reported as part of the US smallpox vaccination program [15]. To improve the cancer selectivity of VV, the viral thymidine kinase (vTK) gene and/or the vaccinia growth factor (VGF) gene can be disrupted/deleted, which leads to the retention of the natural oncolytic activity of VV in cancer cells, but not in normal cells. This is because these two genes are needed for viral replication, and the proteins that they encode are expressed primarily in cancer cells [16]. Further, VV replicates in the cellular cytoplasm and does not integrate its genome in the host nuclei. Its large genome makes the deletion of virulence genes or the insertion of therapeutic genes through genetic engineering relatively simple [17]. It also has broad cancer tropism. For application in OVT, it can be administered intravenously or intratumorally. The benefits of using VV for OVT have been proven in both preclinical and clinical studies, but can still be further enhanced via genetic engineering by substituting the vTk or VGF gene with useful therapeutic genes. Although the efficacy of OV in cancer cells/tissues in clinical settings has previously been proven, studies have been limited to 2–3 cancer types. Therefore, we designed a novel oncolytic virus (NOV) to expand the oncolytic potency of OVs for application in a broad range of cancers.

Cancer is a disease characterized by uncontrolled cellular growth and abnormal vessel formation. Tumor vessels are tortuous, with increased permeability and functional abnormalities. Therefore, vessel normalization is considered an alternative to simple anti-angiogenesis in cancer therapy [18,19]. Angiopoietins (Ang1 and Ang2) and their Tie2 receptor tyrosine kinase have wide-ranging effects in tumors, including angiogenesis, inflammation, tumor vessel normalization, and neovasculogenesis [20,21,22,23]. Tie2 signaling is dynamically involved in normal vessel formation, maintenance, and permeability, which are regulated by the binding of Tie2 to its ligands, Ang1 and Ang2. Ang1 stimulates the tightening of endothelial junctions, resulting in blood vessel stabilization. Ang2 is reported to be overexpressed in tumors. Tumor necrosis factor (TNF)-related apoptosis-inducing ligand (TRAIL), a member of the TNF ligand family, induces apoptosis through death receptors. TRAIL can induce cell death selectively, exhibiting cytotoxicity in many cancer cells but not in normal cells, and thus can be considered a representative anti-cancer therapeutic agent [24,25]. In the present study, NOV was generated by replacing both the VGF and thymidine kinase (TK) regions with Ang1 and TRAIL. We disrupted VGF and/or TK in the VV Wyeth strain genome for the selective infection of tumor cells [16,26]. To enhance the therapeutic activity of the virus, Ang1 and TRAIL were then inserted into the disrupted VGF and TK regions, respectively.

Here, we demonstrate the pan-cancer-specific potency of anti-tumor activity of NOV relative to other comparable recombinant vaccinia viruses and confirm that NOV has superior anti-tumor oncolytic activity in most cancer types (14 cancer cell lines) and in vivo in a colorectal cancer (CRC) syngeneic mouse model.

## 2. Results

### 2.1. Generation of NOV by Insertion of Ang1 and TRAIL into the VGF and TK Regions, Respectively, in the VV Wyeth Strain Genome

**Figure 1:**
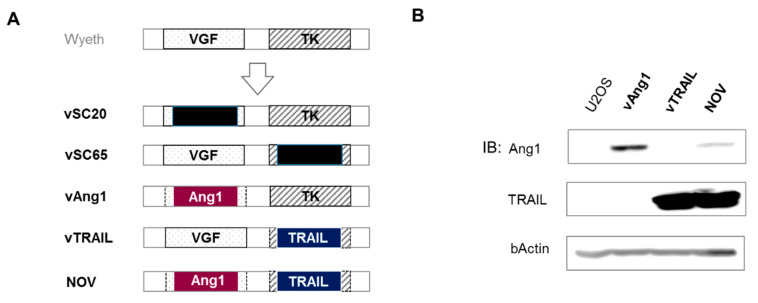
Construction of recombinant novel oncolytic viruses (vAng1, vTRAIL, NOV). (**A**) Schematic diagram of virus construction. Wyeth strain vaccinia virus was used for recombination. vSC20 is vaccinia growth factor (VGF) deleted vaccinia virus. vSC65 is virus thymiding kinase (TK) deleted vaccinia virus. vAng1 was constructed by deletion of VGF and insertion of human angiopoeitin 1 (Ang1); vTRAIL was constructed by deletion of virus TK and insertion of human TNF-related apoptosis-inducing ligand (TRAIL); and NOV was constructed by deleting both VGF and TK and inserting Ang1 and TRAIL, respectively, in their place. (**B**) Western blotting was used to detect TRAIL and Ang1 in U2OS cells 48 h after virus infection (0.1 MOI). Uncropped blots of Figure 1B are shown in Figure S1.

Cancer is a disease characterized by uncontrolled cellular growth associated with abnormal vessel formation. In the past, many researchers have developed drugs to decrease the formation of blood vessels with the aim of killing tumors by blocking their supply of oxygen and nutrients. In contrast, the strategy that we employed in the present study was to design a virus expressing Ang1, which is involved in normal vessel formation, to improve drug delivery and normal tissue reconstruction during the oncolytic process. Direct cancer killing is induced by oncolytic VV itself. In this strategy, the tumor microenvironment may become normal, and the Ang1/Tie2 pathway is critical to this phenomenon [27,28,29]. To improve the pan-cancer selectivity of the virus, VV was genetically engineered to express TRAIL, which can induce cell death selectively in many cancer cells but does not exhibit cytotoxicity in normal cells [24,25]. Therefore, NOV was generated by replacing both the *VGF* and *TK* regions with *Ang1* and *TRAIL*. We disrupted *VGF* and/or *TK* in VV Wyeth strain for the selective infection of tumor cells [16,26]. *Ang1* and *TRAIL* were then inserted into the disrupted *VGF* and *TK* regions, respectively (Figure 1A). vSC20 is VGF deleted VV, and vSC65 is TK deleted VV. vAng1 and vTRAIL are recombinant VVs that are comparable to NOV. vAng1 was generated by replacing *VGF* with *Ang1*, and vTRAIL was generated by replacing *TK* with *TRAIL.* Ang1 and TRAIL expression was confirmed in each virus construct by Western blot analysis using U2-OS (Figure 1B, Figure S1). It was expected that NOV would exhibit enhanced tumor selectivity and the ability to modulate the tumor microenvironment. 

### 2.2. Oncolytic Efficacy of NOV Infection in Various Cancer Cells

**Figure 2:**
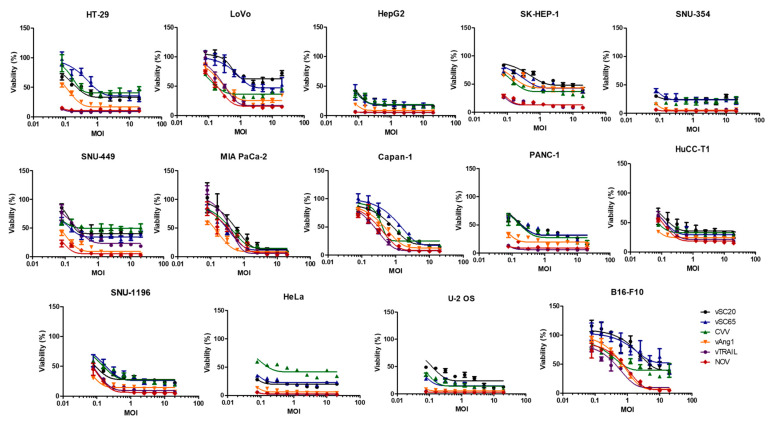
Oncolytic activity of various recombinant vaccinia viruses against different cancer cell lines. Graphs show the viability (%) of two different colon cancer cell lines (HT-29, LoVo), four different hepatocellular carcinoma cell lines (HepG2, Sk-Hep-1, SNU352, SNU449), three different pancreatic cancer cell lines (MIA-PaCa2, CAPAN1, PANC1), two different cholangiocarcinoma cell lines (HuCC-T1, SNU-1196), a cervical cancer cell line (HeLa), an osteosarcoma cell line (U-2 OS), and a murine melanoma cell line (B16-F10) when infected with each virus at different concentrations for 72 h.

To investigate the oncolytic potency of the viruses, 14 cancer cell lines (HT-29, LoVo, HepG2, SK-Hep-1, SNU-354 SNU-449, MIA-PaCa2, Capan-1, PANC-1, HuCC-T1, SNU-1196, HeLa, U-2 OS, B16F10) representing seven types of cancer were infected with WT, vSC20, vSC65, CVV, vAng1, vTRAIL, and NOV for 3 d (Figure 2). CVV was previously constructed, and is a TK-deficient VV with enhanced cancer selectivity [30,31,32,33]. Individual viruses showed high cytotoxicity relative to virus concentration. WT showed dose-dependent oncolytic activity in different cancer cell lines, and this was enhanced by engineering. As expected, NOV showed the highest oncolytic activity, followed by vAng1 and TRAIL, then CVV, and finally vSC20 and vSC65. 

### 2.3. Apoptosis Induced by VV

**Figure 3:**
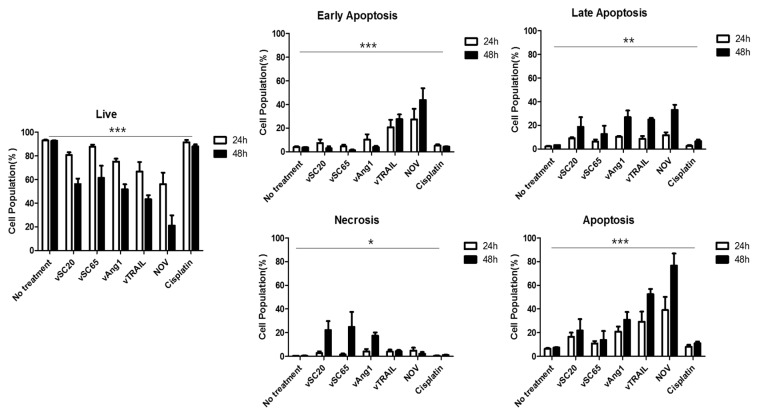
Flow cytometry analysis of HeLa cells treated with each virus or cisplatin. FITC Annexcin V and PI were used for apoptosis and necrosis analysis of tumor cells at 24 h and 48 h after each virus or cisplatin treatment (at concentration of 0.1 MOI and 1 µM, respectively). Live: annexin V^−^ PI^−^ cells, Early Apoptosis: annexin V^+^ PI^−^ cells, Late Apoptosis: annexin V^+^ PI^+^ cells, Necrosis: annexin V^−^ PI^+^ cells. Apotosis: annexin V^+^ cells (early+late apoptosis). *** *p* < 0.0001, one-way ANOVA ** *p* < 0.01, one-way ANOVA * *p* < 0.05, two-way ANOVA.

We then investigated the apoptotic effects of our engineered VVs. HeLa cells were treated with PBS (no treatment control, NT), engineered VVs (vSC20, vSC65, CVV, vAng1, vTRAIL, and NOV at 0.1 MOI), or 1 µM cisplatin. After 24 h and 48 h, cells were stained with annexin V-FITC and PI for apoptosis and necrosis analysis (Figure 3). As expected, the live cell population was decreased by our engineered VVs; NOV produced the strongest effect, followed by vAng1 and vTRAIL, and finally vSC20 and vSC65. Cisplatin treatment had comparatively little effect on the live cell population. Treatment with the engineered VVs induced a higher percentage of annexin V+/PI− (early apoptotic) and annexin V+/PI+ (late apoptotic) cells than NT or cisplatin treatment. NOV treatment resulted in the highest percentage of apoptotic cells. Infection with NOV at 0.1 MOI resulted in significant cell apoptosis (39.03% at 24 h and 76.71% at 48 h post infection) compared to NT (6.40% at 24 h and 7.37 % at 48 h), vSC20 (16.59% at 24 h and 12.90% at 48 h), vSC65 (10.84% at 24 h and 13.79% at 48 h), vAng1 (29.29% at 24 h and 30.84% at 48 h), vTRAIL (30.29% at 24 h and 52.44% at 48 h), and cisplatin (7.92% at 24 h and 10.92% at 48 h). Interestingly, necrotic cell populations were 0.40% at 24 h and 0.52% at 48 h under NT, 2.67% at 24 h and 22.12% at 48 h under vSC20 treatment, 1.50% at 24 h and 24.80% at 48 h under vSC65 treatment, 4.16% at 24 h and 17.4% under vAng1 treatment, 3.92% at 24 h and 4.30% at 48 h under vTRAIL treatment, 4.82% at 24 h and 2.14% at 48 h under NOV treatment, and 0.55% at 24 h and 1.13% at 48 h under cisplatin treatment. Interestingly, the necrotic cell populations under vSC20 and vSC65 treatment were higher than the apoptotic cell population in cellular death. The apoptotic cell population was higher under treatment with other viruses, however; it was highest under NOV treatment, followed by vTRAIL, and finally vAng1. Based on these results, Ang1 incorporation into VV is likely to support cancer cell apoptosis with oncolytic viral activity (vSC20 < vAng1). As expected, TRAIL incorporation induced cancer cell apotosis. Cisplatin also induces apoptotic death (~12% dead cells = ~10% apoptosis), but not effective in killing cancer cells. Of all of the engineered viruses, NOV induced the most cell death via cancer cell apoptosis (~80% dead cells = ~80% apoptosis, Figure 3 bottom left and right). Therefore, NOV would give the highest and efficient apoptosis-induced cancer killing activity.

### 2.4. Cancer-Specific Apoptosis and Anti-Tumor Immunity Induced in Tumors after Intraperitoneal Injection of Engineered VVs in Mouse Colon Cancer Model

**Figure 4:**
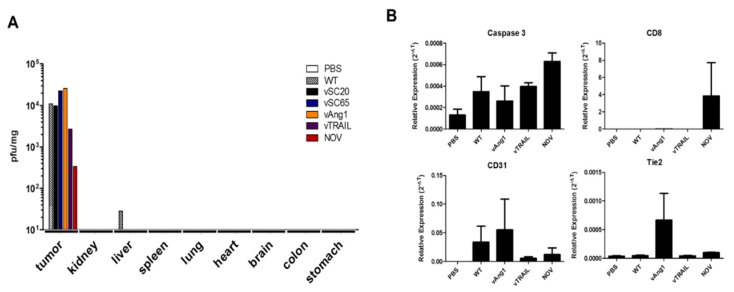
In vivo cancer selectivity of engineered vaccinia viruses in a mouse colon cancer model. (**A**) Biodistribution results 1 week after intraperitoneal injection of viruses in HT-29-bearing mice, showing that the vaccinia virus (Wyeth strain) selectively targets tumor cells. This can be enhanced by recombination (vTK or VGF deletion). (**B**) Caspase 3, CD8, CD31, and Tie2 mRNA expression in the harvested tumor mass 1 week after intraperitoneal injection in CT26-bearing mice.

To evaluate whether the cancer killing activity and induction of apoptosis described above is cancer-specific and biosafe in vivo, biodistribution studies were performed with tumor and normal organ tissues from a mouse colon model 1 week after intraperitoneal infection with VVs. To test the biodistribution of intraperitoneally injected VVs in each organ, HT-29-xenograft nude mice were used (Figure 4A). Viral titration results (pfu/mg) showed exclusive and robust infection of tumors by engineered VVs and even WT, with negligible effects on normal tissues. The NOV and vTRAIL seems to have attenuated titration results in vivo in comparison to the other viruses, which is maybe because it expresses both two genes. It is also likely that TRAIL expressed by VV has some effect on this because of its killing effect on cancer tissue (thereby leading to harvesting less live cancer tissues), while Tk/VGF deletion gives more cancer selectivity. To investigate the cancer-specific apoptosis and anti-tumor activity induced in tumors after intraperitoneal virus injection, CT26-bearing BALB/c mice were used. Interestingly, we found that both of caspase 3 and CD8 expression were upregulated noticeably in tumor tissues after systemic injection of NOV (Figure 4B, top panel). The expression of CD31 and Tie2 is of endothelial origin [34]. The contribution of vAng1 to normal vessel formation as well as to tumor cell apoptosis was confirmed by CD31 and Tie2 expression (Figure 4B, bottom panel), while NOV has somewhat suppression effect of both CD31 and Tie2 expression, compared to vAng1, maybe due to simultaneous TRAIL expression [35] (Figure 1B). The expression of Tie2 was higher in NOV-treated cells than in WT- and vTRAIL-treated cells. Additionally, caspase 3 and CD8 showed higher activation in NOV-treated cells than in cells treated with other engineered VVs. These results confirm that the oncolytic efficacy of NOV is cancer-specific in vivo. The presence of both Ang1 and TRAIL is advantageous as it leads to the induction of cancer-specific apoptosis and anti-tumor immunity with supportive normal vessel construction/stabilization. Therefore, NOV exhibits both anti-tumor activity and biosafety. 

### 2.5. NOV Has Highest Therapeutic Efficacy of All VVs in CRC Syngeneic Mouse Model

**Figure 5:**
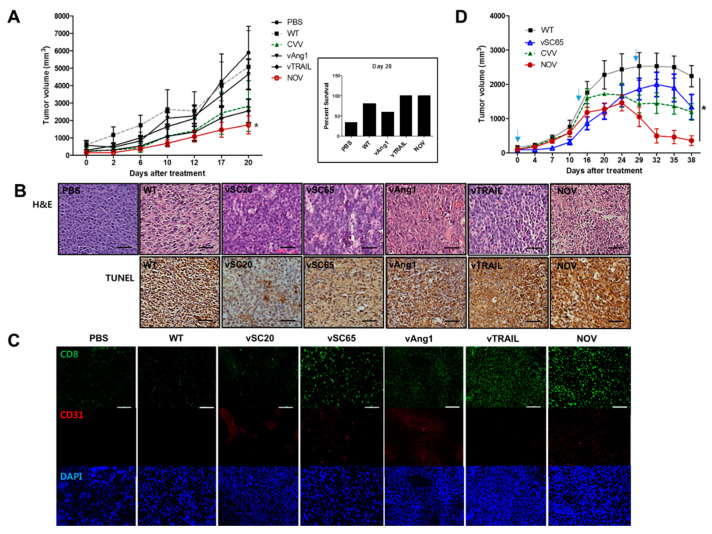
Therapeutic efficacy of NOV in a colorectal cancer (CRC) syngeneic mouse model. (**A**) Tumor growth in a CT26-bearing mouse cancer model treated with PBS or viruses (left). Percent survival of mice in each treatment group (right). *N* = 5. * *p* < 0.5 (*t*-test, NOV vs. WT). (**B**) Hematoxylin and eosin (H&E) staining and transferase dUTP nick end labeling (TUNEL) results for tumor tissues of mice treated with PBS and viruses. Scale bar = 10 µm (**C**) Immunofluorescence staining of CD8+ cells (CD8), vascular cells (CD31) and nuclei (DAPI). Scale bar = 10 µm (**D**) Therapeutic efficacy of each virus in the CT26-induced tumor model. When the tumor reached 100 mm^3^, each virus treatment was started. The treatment schedules were noted with downwards arrows. *N* = 4. * *p* < 0.5, one-way ANOVA.

BALB/c mice were used to establish a CRC syngeneic mouse model (with CT26 cells). To investigate the effect of each engineered VV on survived cancer cell growth in vivo, CT26 cells were treated with PBS or viruses (MOI = 0.1) and harvested after an additional 2 h in culture. Live (survived) cells from each group were counted and injected subcutaneously (1 × 10^6^ cells/100 µL) into the mice, and tumor growth from the injected cells was subsequently examined. Mice were categorized into six groups (PBS, WT, CVV, vAng1, vTRAIL, NOV), with five mice per group. A slower burden in each virus treatment group was observed compared with the PBS group (PBS < WT < vAng1 < CVV < vTRAIL < NOV). The NOV group showed the highest attenuated tumor growth (Figure 5A left, *p* < 0.5, *t*-test vs. WT group). At 28 d after injection, the percent survival in each group was 33.4% in PBS, 60% in WT, 40% in vSC20, 40% in vSC65, 80% in CVV, 60% in vAng1, 100% in vTRAIL, and 100% in NOV. Additionally, the survival rate in all virus-treated groups was higher than in the PBS group (Figure 5A left, PBS < vAng1 < WT < CVV < vTRAIL < NOV).

To determine whether tumor growth is inhibited via apoptosis induced by NOV, we performed H&E staining and TUNEL analysis for histomorphologic analysis and detection of apoptosis, respectively (Figure 5B). The result showed that the oncolytic efficacy of NOV is largely due to the induction of cell apoptosis. Therefore, the incorporation of TRAIL into engineered VVs increases efficacy, and efficacy can be further increased by the presence of Ang1. We also performed immunological analysis of tumor sites by conducting immunofluorescence staining of CD8 (Figure 5C). It is worth noting that an influx of CD8+ cells into the tumor microenvironment is an important mechanism for killing cancer cells. The result of immunofluorescence staining showed induced CD8+ T lymphocyte infiltration in the vTRAIL and NOV groups. Further immunofluorescence staining showed increased CD31 density in the NOV group, confirming the more normal distribution of vascularization in NOV than in Ang1. Therefore, the ability to normalize tumor vessels contributes to the therapeutic efficacy of NOV. 

To prove, the oncolytic efficacy of the selected VVs (WT, pSC65, CVV, and NOV) was investigated in vivo. CT26 cells (1 x 10^7^)/site was injected subcutaneously into the left and right flank of each mouse. Mice were categorized into the each VV group with four mice per group. When the tumor volume reached ~100 mm^3^, 1 × 10^6^ pfu of each virus were injected intratumorally to the corresponding groups once per two weeks. The therapeutic efficacy of engineered VVs (NOV > CVV > vSC65 > WT) was confirmed by the enhanced tumor regression, which is apparent after 1-month treatment (Figure 5D), which is maybe because the attenuated replication of engineered VVs with a burden of harboring therapeutic gene to be expressed. 

## 3. Discussion

Although VV was used as a smallpox vaccine until the disease was declared eradicated by the WHO in 1980, its exact origin is unknown. Its ability to kill cancer cells was first reported by Levaditi C. and Nicolau S. in the Annals of the Pasteur Institute in 1923 [36]. VV is a double-stranded DNA virus that infects almost all mammalian cells [37]. An important advantage of VV engineering is that up to 25 kb of the desired therapeutic gene to be expressed can be inserted into its viral genome without loss of its capacity to infect and replicate within the host cancerous cells [17,38,39]. The anti-cancer potential of VV has been emphasized in preclinical trials, and various genes have been inserted into its genome to improve its oncolytic properties [29,40]. The genetic modification of recombinant VVs not only increases their tumor affinity, but also enhances their functionality through the expression of proteins with specific anti-tumor activities. Recent reports suggest that stably changing structural and functional abnormalities in cancer vessels using Tie2 active antibodies (ABTAA), which act specifically on cancer vascular endothelial cells, can normalize cancer vessels and inhibit cancer growth and metastasis [22]. It has been reported that some VVs have an effect on tolerance to apoptosis, which limits the efficiency of tumor cell lysis [40]. Although the results of our experiments with engineered VV have shown positive in stem cell-like colon cancer [30], metastatic hepatocellular carcinoma [31], and cholangiocarcinoma [33], their tumor cell lysis efficiency should be increased including more types of tumors. In the present study, we developed NOV, which exhibits the dual advantages of cancer selectivity and normal vessel reconstructive properties as a result of the expression of *TRAIL* and *Ang1* in the place of *vTk* and *VGF*, respectively (Figure 1).

Our virus design strategy involved utilizing Ang1 to improve drug delivery as well as normal tissue reconstruction during the oncolytic process. Meanwhile, cancer cells are killed directly by oncolytic VV. Under this strategy, it is possible for the tumor microenvironment to become normal; the Ang1/Tie2 pathway is critical in this process [27,28,29]. To improve the pan-cancer killing selectivity of VV, TRAIL was used because it can induce death selectively in many cancer cells but does not show cytotoxicity in normal cells [24,25]. The pan-cancer-specific oncolytic potency of NOV was confirmed in different human and mouse cancer cell lines (colon, liver, pancreas, cholangiocarcinoma, cervical cancer, osteosarcoma, and melanoma). NOV showed the highest toxicity of several comparable recombinant viruses, including vSC20, vSC65, vAng1, vTRAIL, and WT (Figure 2). VV treatment directly induced early apoptosis in tumors within 24 h, and this effect was enhanced with further engineering; VGF and Tk deletion with Ang1 and TRAIL insertion in NOV was the most effective modification, followed by Tk deletion and TRAIL insertion in vTRAIL, then VGF deletion and Ang1 insertion in vAng1, and finally VGF deletion in vSC20 or Tk deletion in sSC65. Meanwhile, treatment with the conventional anti-cancer drug cisplatin did not induce apoptosis (Figure 3). This apoptosis was cancer-specific; VV (Wyeth strain) administered via intraperitoneal injection selectively infected tumor cells and induced caspase 3 and CD8 expression in the tumor. This was also enhanced by further engineering, most strongly in NOV, followed by vTRAIL, then finally vAng1 (Figure 4). A virus-treated CT26 mouse colon cancer syngeneic model showed attenuated tumor growth, which was in accordance with the results of percent survival measurement, CD8 expression analysis, and TUNEL staining with advanced genetic engineering (vAng1 < vTRAIL < NOV) (Figure 5). These results indicate that the oncolytic efficacy of NOV is primarily a result of the induction of cell apoptosis. Therefore, the incorporation of TRAIL into engineered VVs increases their efficacy, and efficacy can be increased still further by the incorporation of Ang1. An influx of CD8+ cells into the tumor microenvironment is an important mechanism for killing cancer cells; induced CD8+ T lymphocyte infiltration was highest in the NOV group.

In summary, NOV successfully induced cancer-targeted apoptosis and anti-tumor immunity, and may constitute a highly advantageous therapeutic agent for next-generation solid tumor virotherapy with pan-cancer-specific oncolytic activity and high biosafety. 

## 4. Materials and Methods 

### 4.1. Cell Culture and Reagents 

Cell lines were obtained from the Korean Cell Line Bank (Seoul, Korea) and the ATCC (Manassas, VA, USA). The cells were cultured in Roswell Park Memorial Institute (RPMI) 1640 medium, which was supplemented with 10% fetal bovine serum and 100 U/mL penicillin and streptomycin under standard conditions of 37 °C, 5% carbon dioxide, and a humidified atmosphere. All culture media and supplements were obtained from Welgene (Daegu, South Korea). The anti-cancer drug cisplatin was purchased from Selleckchem (Houston, TX, USA). All antibodies were purchased from Abcam (Cambridge, UK). FITC Annexin V assay kits were from BD Biosciences Korea (Seoul, South Korea)

### 4.2. Western Blot 

Protein extraction was performed with PRO-PREP^TM^ Protein Extraction Solution (iNtRON Biotechnology, Seoul, South Korea) according to the manufacturer’s recommendations. Protein concentration was determined using Bradford Reagent (Bio-Rad Laboratories Inc., Hercules, CA, USA). Equal amounts of proteins (100 µg) from the clarified lysates were separated by 10% sodium dodecyl sulfate–polyacrylamide gel electrophoresis (SDS-PAGE) and transferred to a nitrocellulose membrane with 0.45 mm pores (Whatman, Marlborough, MA, USA). The membranes were blocked with 5% BSA in PBS with Tween 20 (PBST) for 1 h at room temperature and then reacted overnight with primary antibodies against angiopoietin 1 antibody (Abcam; 1:1000), TRAIL antibody (Abcam; 1:1000), and anti-beta-actin antibody (Abcam; 1:1000), and for 2 h at room temperature with secondary anti-rabbit immunoglobulin G (IgG) horseradish peroxidase (HRP)–linked antibody (1:1000), and antimouse IgG HRP-linked antibody (1:1000). Immunoreactive bands were visualized using an enhanced chemiluminescence WEST-Queen Western Blot Detection System (iNtRON).

### 4.3. Cell Proliferation (Cytotoxicity) Assay 

Cells were seeded at 10,000 cells per well in 96-well plates. After 1 d, the cells were treated for 2 h with various OVs (vSC20, vSC65, CVV, vAng, vRAIL, NOV) at different multiplicities of infection (MOIs) in serum-free media. The media were then replaced with normal culture media. At 72 h after treatment, cell viability was assessed by WST assay with EzCytotox (iTSBiO, Seoul, Korea) in accordance with the manufacturer’s instructions. The absorbance of each sample was measured using a microplate reader (TECAN, Männedorf, Switzerland) at 450 nm. The reference wavelength was 680 nm. The viability of cells with no treatment (0 MOI or µM, control) was set to 100%, and each group’s corresponding viability was expressed as a percentage of viability compared with that of the control group. 

### 4.4. Apoptosis and Necrosis Analysis 

Cells seeded in 6-well plates were treated with PBS (control), each virus (vSC20, vSC65, vAng, vRAIL, NOV), or cisplatin, then incubated in a CO_2_ incubator. After 24 h and 48 h, cells were harvested by trypsinization, washed with PBS, and suspended in PBS containing 2% BSA. Cells were then suspended in 100 μL binding buffer and 4 μL FITC Annexin V (BD sciences, San Jose, CA, USA), and 4 μL propidium iodide was added to the cell suspension. The suspension was gently vortexed and incubated for 15 min at room temperature (25 °C) in the dark. Apoptosis and necrosis were analyzed by flow cytometry using the Navios flow cytometer (Beckman Coulter, Pasadena, CA, USA). The FACS data were analyzed using Kaluza Analysis Software 2.1 (Beckman Coulter).

### 4.5. Real-Time Polymerase Chain Reaction

Total RNA was extracted using TRIzol reagent (Life Technologies, Carlsbad, CA, USA) in accordance with the manufacturer’s instructions. RNA purity was verified by measuring the 260/280 absorbance ratio. The first strand of complementary DNA (cDNA) was synthesized with 2 μg total RNA using the RH(−) RT Synthesis Kit (iNtRON Biotechnology). Two microliters of cDNA were used for each polymerase chain reaction (PCR) mixture containing SYBR Green qPCR mix (Roche, Basel, Switzerland). Real-time PCR was performed using the LightCycler 96 Real-Time PCR System (Roche). The reaction conditions were as follows: 45 cycles of amplification at 95 °C for 10 s, at 60 °C for 10 s, and at 72 °C for 10 s. The relative mRNA expression of the selected genes was normalized to beta-actin and quantified using the ΔCt method. The primers used are listed in Table 1.

### 4.6. Biodistribution

Two or three 5-week-old nude male mice per each group were given subcutaneous injections of 1 × 10^7^ HT-29 human cancer cells in 100 µL PBS (*n* = 2 for PBS group, *n* = 3 for each virus group). At 7 d after injection of HT-29 or CT26 cells, the mice were given intraperitoneal injections of PBS or 1 × 10^7^ pfu WT, vSC20, vSC65, vAng, vTRAIL, or NOV in 100 µL PBS. Then, 7 d post infection, mice were sacrificed and whole sections of the stomach, colon, brain, heart, lung, spleen, liver, kidney, and tumor were taken and homogenized in PBS. Viruses in these tissues were titrated using U2-OS cells, and viral yield was calculated per milligram of tissue (pfu/mg). BALB/c mice and CT26 cells were used to measure the mRNA expression of caspase 3, CD8, CD31, and Tie2 in the tumor tissue 7 d after intraperitoneal injection of each virus.

### 4.7. CRC Syngeneic Mouse Model

All mice were maintained in accordance with the Institutional Animal Care and Use Committee–approved protocols of Pusan National University (Busan, South Korea; PNU-2019-2128). BALB/c mice were used to establish a CRC syngeneic mouse model with CT26 cells. We then cultured CT26 cells for 7 d, treated them with PBS or each virus (MOI = 0.1), and harvested them after an additional 2 h in culture. Live cells (1 × 10^6^ cells/100 µL) from each group were injected subcutaneously into the mice, and tumor growth from the injected cells was examined after treatment. Tumor growth was measured twice per week using digital calipers. The following formula was used to calculate tumor volume: tumor volume (mm^3^) = (length × width^2^)/2. For the therapeutic efficacy test of each virus (Figure 5D), CT26 (1 × 10^7^ cells/100 µL) was injected subcutaneously into the left and right flank of the mice. When tumor volume reaches around 100 mm^3^, mice were divided into 4 groups (4 mice/ group). Then, 100 µL of 1 × 10^6^ plaque-forming units (pfu) of the virus were injected intratumorally to the corresponding mice groups once per two weeks.

### 4.8. H&E Staining, TUNEL Analyses, and Immunofluorescent Assay 

Tumor tissues from mice in each group were fixed in 10% formalin solution and embedded in paraffin (FFPE). Cross-sections of each sample were stained with hematoxylin and eosin (H&E) for histomorphologic analysis and transferase dUTP nick end labeling (TUNEL) to detect apoptosis. For immunofluorescence staining, the FFPE tumor sections were permeabilized with 0.1% Triton X-100 and nonspecific binding was blocked with 5% normal goat serum (Invitrogen, Thermo Fisher Scientific Inc., Waltham, MA, USA). Immunofluorescence staining was performed for CD8 and CD31 with labeled secondary antibodies, followed by counterstaining with DAPI for nuclei. Fluorescence microscopy (Nikon Eclipse Ni, Melville, NY, USA) was employed to examine stained samples.

## 5. Conclusions

A novel oncolytic vaccinia virus (NOV) was demonstrated with the dual advantages of cancer selectivity and normal vessel reconstructive activity by replacing the viral thymidine kinase (vTk) and vaccinia growth factor (VGF) genes with genes encoding TNF-related apoptosis-inducing ligand (TRAIL) and angiopoietin 1 (Ang1). NOV successfully induced cancer-targeted apoptosis and anti-tumor immunity, and may constitute a highly advantageous therapeutic agent for next-generation solid tumor virotherapy with pan-cancer-specific oncolytic activity and high biosafety.

## 6. Patents

There are patents resulting from the work reported in this manuscript: Novel Cancer Favored Oncolytic Virus and Use Thereof (Korean Patent No. 101605176), Novel Oncolytic Virus, Method for Preparing, and Uses Thereof (Korean Patent No. 101845739)

## Figures and Tables

**Table 1:** Primers used in this study.

Name	Sequence (5′–3′)
Mouse Caspase 3 Forward (mCas3-F)	GGGCCTGTTGAACTGAAAAA
Mouse Caspase 3 Reverse (mCas3-R)	CCGTCCTTTGAATTTCTCCA
Mouse CD8 Forward (mCD8a_F)	CAGAGACCAGAAGATTGTCG
Mouse CD8 Reverse (mCD8a_R)	TGATCAAGGACAGCAGAAGG
Mouse CD31 Forward (mCD31-F)	TGCAGGAGTCCTTCTCCACT
Mouse CD31 Reverse (mCD31-R)	ACGGTTTGATTCCACTTTGC
Mouse Tie2 Forward (mTie2-F)	AAGCATGCCCATCTGGTTAC
Mouse Tie2 Reverse (mTie2-R)	GTAGGTAGTGGCCACCCAGA
Mouse β-Actin Forward (BAT-Fw)	GTCCCTCACCCTCCCAAAAG
Mouse β-Actin Reverse (BAT-Re)	GCTGCCTCAACACCTCAACCC

## Supplementary Materials

The following are available online at https://www.mdpi.com/2072-6694/12/5/1070/s1, Figure S1: Western blots to detect Angiopoietin 1, TRAIL, and beta-actin in U2OS cells 48 h after virus infection (0.1 MOI). Densitometry readings of each corresponding bands are shown in the graph below.
