# Supplementary material for: Novel Oncolytic Virus Armed with Cancer Suicide Gene and Normal Vasculogenic Gene for Improved Anti-Tumor Activity
Source: Cancers (Basel). 2020 Apr 25;12(5):1070. doi: 10.3390/cancers12051070 (PMC7281019; doi:10.3390/cancers12051070)
Supplement: Supplementary file 1 [file cancers-12-01070-s001.pdf]

Supplementary Materials: Novel Oncolytic Virus Armed with Cancer Suicide Gene and Normal Vasculogenic Gene for Improved Anti-Tumor Activity

Su-Nam Jeong and So Young Yoo

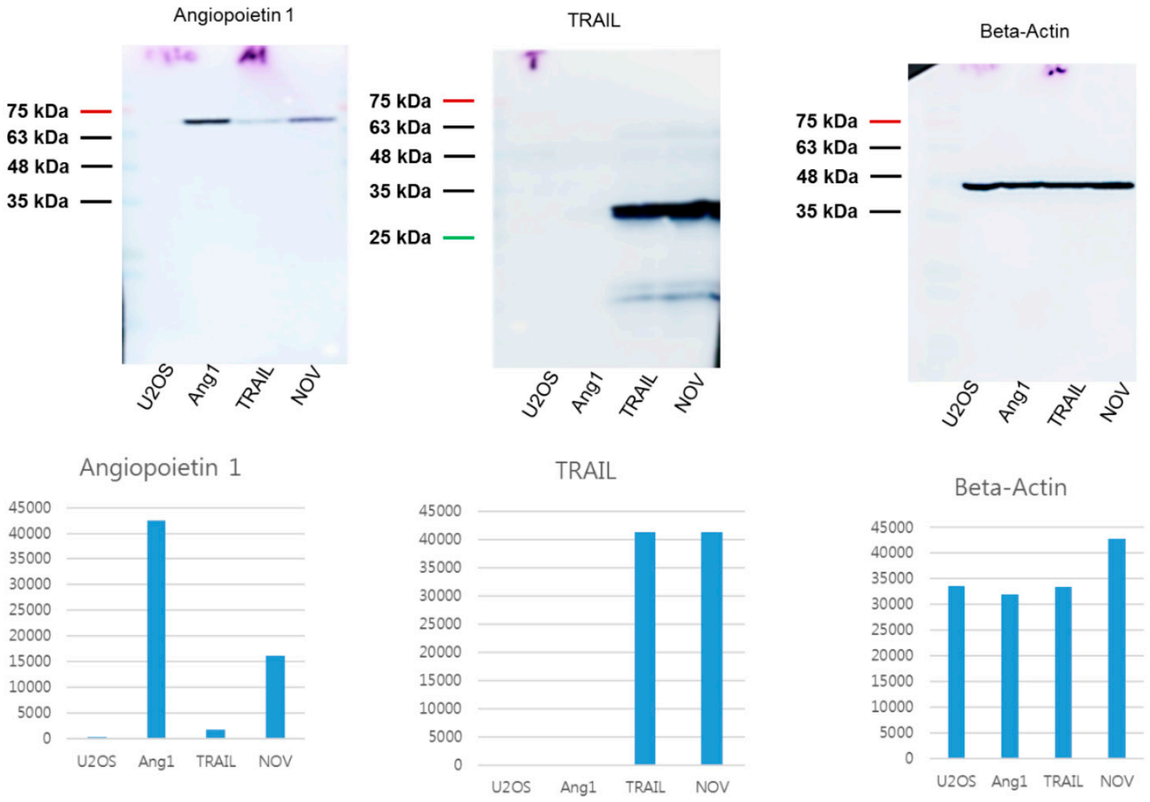

Figure S1. Western blots to detect Angiopoietin 1, TRAIL, and beta-actin in U2OS cells 48 h after virus infection (0.1 MOI). Densitometry readings of each corresponding bands are shown in the graph below.

© 2020 by the authors. Licensee MDPI, Basel, Switzerland. This article is an open access article distributed under the terms and conditions of the Creative Commons Attribution (CC BY) license (http://creativecommons.org/licenses/by/4.0/).
